# The D_1_ dopamine receptor agonist, SKF83959, attenuates hydrogen peroxide-induced injury in RGC-5 cells involving the extracellular signal-regulated kinase/p38 pathways

**Published:** 2012-12-01

**Authors:** Guang-Yu Li, Ting Li, Bin Fan, Yong-Chen Zheng, Tong-Hui Ma

**Affiliations:** 1 Department of Ophthalmology, Second Hospital of JiLin University, Changchun, China; 2 State Key Laboratory, Second Hospital of JiLin University, Changchun, China

## Abstract

**Purpose:**

Oxidative stress is widely implicated in the death of retinal ganglion cells associated with various optic neuropathies. Agonists of the dopamine D_1_ receptor have recently been found to be potentially neuroprotective against oxidative stress–induced injury. The goal of this study was to investigate whether SKF83959, a next-generation high-affinity D_1_ receptor agonist, could protect retinal ganglion cell 5 (RGC-5) cells from H_2_O_2_-induced damage and the molecular mechanism involved.

**Methods:**

We examined expression of the D_1_ receptor in RGC-5 cells with reverse-transcription–PCR and immunoblotting and assessed neuroprotection using propidium iodide staining and the 3-(4,5-dimethylthiazol-2-yl)-2,5-diphenyltetrazolium bromide assay. In addition, we monitored the activation and involvement of members of mitogen-activated protein kinase family, extracellular signal-regulated kinase (ERK), p38 and c-Jun NH_2_-terminal kinase, with western blot and specific inhibitors.

**Results:**

We found that the D_1_ receptor was expressed in RGC-5 cells, but the sequence analysis suggested this cell line is from mouse and not rat origin. SKF83959 exhibited a remarkable neuroprotective effect on H_2_O_2_-damaged RGC-5 cells, which was blocked by the specific D_1_ receptor antagonist, SCH23390. ERK and p38 were activated by SKF83959, and pretreatment with their inhibitors U0126 and SB203580, respectively, significantly blunted the SKF83959-induced cytoprotection. However, the specific c-Jun NH_2_-terminal kinase inhibitor, SP600125, had no effect on the SKF83959-induced protection.

**Conclusions:**

We conclude that SKF83959 attenuates hydrogen peroxide–induced injury in RGC-5 cells via a mechanism involving activation of the ERK and p38 pathways and the D_1_ receptor is a potential molecular target for developing neuroprotective drugs.

## Introduction

Oxidative stress is widely implicated in the death of retinal ganglion cells (RGCs) associated with various ocular neurodegenerative disorders, such as glaucoma, Leber hereditary optic neuropathy, ischemic optic neuropathy, and traumatic optic neuropathy [1-4]. Studies have demonstrated that under oxidative stress, reactive oxygen species (ROS) including free radicals such as superoxide (O^2−^), hydroxyl radical (HO^−^), and hydrogen peroxide (H_2_O_2_) are generated at high levels inducing cellular damage and even cell death [5]. Elevated levels of ROS may cause increased permeability of the blood–retina barrier, tubulin alterations, and perturbation in synaptic transmission [6-8]. Emerging evidence further suggests that under pathologic conditions, excessive amounts of ROS induced by oxidative stress can modify proteins, lipids, and DNA to alter their functions and activate signaling pathways resulting in death of retinal neurons [9].

Activation of the dopamine D_1_ receptor was recently found to be potentially neuroprotective against oxidative-stress damage in retinal neurons including RGCs [10]. Dopamine is the main catecholamine found in the retina of most species, which is synthesized from the L-amino acid tyrosine [11]. Dopamine has been suggested to play a developmental role in the embryonic retina [12]. Based upon structural and pharmacological similarities, the dopamine receptor family includes five members, which are divided into two subfamilies: the D_1_-like family, comprising D_1_ and D_5_ receptors, and the D_2_-like family, containing D_2_, D_3_, and D_4_ receptors [13]. D_1_-like receptors have high structural homology across species between amino acids 445 and 488 [14]. In addition, D_1_-like receptors do not contain introns in their protein coding regions decreasing the possibility of observing receptor variants [15]. The protein structure of D_1_-like dopamine receptors consists of putative transmembrane domains, potential glycosylation sites in the first extracytoplasmic loop, and a carboxyl terminal tail [16]. Upon stimulation, D_1_-like receptors trigger signal transduction cascades mediated through adenylyl cyclase or phosphoinositide metabolism accompanied by subsequent enhancement of multiple downstream kinase cascades [15]. In clinical settings, agonists of the D_1_ receptor have been used in treating Parkinson disease since dihydrexidine (DHX), the ﬁrst high-afﬁnity D_1_ agonist with full intrinsic activity, was developed [17]. During Parkinson disease therapy, another important, possible benefit of using D_1_ receptor agonists was found: neuroprotection [18]. Moreover, many studies show that activation of the D_1_ receptor also provides excellent ocular neuroprotection [19,20]. Kipnis et al. found that the selective dopamine receptor D_1_ agonist, SKF38393, a first-generation D_1_ receptor agonist, protected primary cultures of fetal rat retinal cells from glutamate neurotoxicity [21]. Subsequently, Maher et al extended the protective effects of SKF-38393 by demonstrating its ability to also protect retinal ganglion cells (RGC-5) from oxidative stress-mediated injury induced by either glutamate plus buthionine sulfoximine (BSO), tert-butyl peroxide (t-BOOH), or H_2_O_2_ treatments [22].

Members of the mitogen-activated protein kinase (MAPK) family play a critical role in oxidative stress–induced neuronal death since MAPK signaling cascades involve highly conserved serine/threonine kinases connecting cell surface receptors to regulatory targets in response to oxidative stress [23]. The MAPK signaling pathways mainly occur through activation of three kinase subfamilies: the stress-activated protein kinases (c-Jun NH_2_-terminal kinase [JNK]), the p38 kinases, and the extracellular signal-regulated kinases (ERK) [24]. Activation of MAPKs is through upstream kinases, including mitogen-activated protein kinase kinase 1 and 2 (MKK1/2), MKK3/6, and MKK4/7, which can reversibly phosphorylate threonine and tyrosine residues of the TXY motif in the catalytic domain. ERK and p38 normally are activated by MKK1/2 and MKK3/6, respectively, whereas JNK is activated by MKK4/7 [25]. Once activated, MAPKs phosphorylate several cellular substrates to propagate signaling cascades leading to many forms of cellular responses, including proliferation, differentiation, and death [26]. Although prior studies have explored the molecular basis of neuroprotection offered by D_1_ receptor agonists in various neuronal cells [27-30], the exact signaling pathway elicited by D_1_ receptor stimulation of RGCs is still unclear. Whether D_1_ receptor agonists protect RGCs against oxidative stress–induced injury through regulating MAPK pathways still needs to be elucidated.

The goal of this study was to determine whether SKF83959, a next-generation high-affinity D_1_ receptor agonist [31], protected RGCs against oxidative stress-dependent damage. To test this hypothesis, the retinal ganglion cell line, RGC-5, was used as an in vitro model to determine the molecular basis of SKF83959 protection. The results indicated that SKF83959 protects RGC-5 cells from H_2_O_2_-induced injury in an ERK- and p38-dependent fashion.

## Methods

### Chemicals and reagents

Cell culture media and additives were obtained from Hyclone (Beijing, China), and plastic cultureware was supplied by DingGuo BioTech (Beijing, China). The rabbit anti-D_1_ receptor, anti-p-ERK, anti-p38, and anti-p-JNK monoclonal antibodies were purchased from Bioworld (Hong Kong, China). The mouse anti-β-actin monoclonal antibody was obtained from Chemicon (Watford, UK). The reverse-transcription–PCR (RT–PCR) commercial kit and DNA marker were purchased from Takara (Dalian, China). The anti-rabbit immunoglobulin G and all the other reagents and inhibitors were purchased from Sigma-Aldrich (Shanghai, China).

### Cell culture

RGC-5 cells were ordered from the American Type Culture Collection (ATCC, Manassas, VA) and grown in Dulbecco’s modified Eagle’s medium (DMEM; Hyclone, Beijing, China) supplemented with 10% heat-inactivated fetal bovine serum, 100 U/ml penicillin, and 100 mg/ml streptomycin in a humidified atmosphere of 95% air and 5% CO_2_ at 37 °C. The doubling time of the cells was approximately 20 h under these conditions, and the cells were generally passaged by trypsinization at a ratio of 1:6 every 3 to 4 days.

### Cell viability assays and propidium iodide staining

Cell viability was assessed using a 3-(4,5-dimethylthiazol-2-yl)-2,5-diphenyltetrazolium bromide (MTT) reduction assay modified from that described by Mosmann et al. [32]. MTT was added to each well at a final concentration of 0.5 mg/ml in minimum essential medium (MEM) that lacked serum and phenol red and incubated for 1 h at 37 °C. Reduced MTT (blue formazan product) was solubilized with dimethyl sulfoxide, and the absorbance was determined using an automated microplate reader (Titertek Plus MS212; ICN Flow, Thame, UK) with a 570 nm test wavelength and a 690 nm reference wavelength. Concentrations of U0126, SB203580, and SP600125 were preliminarily screened to assess the effects of drug alone toxicity in the cultures to select an appropriate non-toxic concentration (data not shown). For propidium iodide (PI) staining, the cells were first cultured in a 24-well plate for 24 h. After being treated with 500 μM H_2_O_2_ for 5 h, the cells were then treated with the PI solution at a final concentration of 2 μg/ml and incubated for 10 min at room temperature. The PI-positive cells were visualized on an inverted fluorescence microscope (Leica; Berlin, Germany).

### RNA extraction and real-time polymerase chain reaction amplification

Total RNA was extracted from RGC-5 cells using the TRIzol method (Takara). RNA samples were stored at −80 °C. The RNA concentration was determined spectrophotometrically by measuring absorbance at 260 nm and with agarose gel electrophoresis. RT–PCRs were performed with the RT–PCR commercial kit (Takara), using 1 µg of total RNA treated with DNase. Reactions were incubated at 45 °C for 45 min and 94 °C for 2 min, and then through 30 cycles of 94 °C for 30 s, 55 °C for 1 min, and 72 °C for 30 s, with a final extension incubation of 7 min at 72 °C. Amplification product was analyzed with agarose gel electrophoresis and sequenced from Takara. Analyses and comparisons of the resulting sequence were performed using the BLAST tool (NCBI online).

### Western blotting

RGC-5 cells were sonicated in protein lysate buffer (20 mm Tris-HCl, pH 7.4, 25 °C, 2 mm EDTA, 0.5 mm ethyl glycol tetraacetic acid [EGTA], 1 mm dithiothreitol, 50 mg/ml leupeptin, 50 mg/ml pepstatin A, 50 mg/ml aprotinin, and 0.1 mm phenylmethylsulfonyl fluoride). The bicinchoninic acid assay (BCA) method was used to estimate protein concentrations [33]. An equal amount (20 µg) of cell lysate was dissolved in sample buffer (62.5 mm Tris-HCl, pH 7.4, 4% sodium dodecyl sulfate, 10% glycerol, 10% β-mercaptoethanol, and 0.002% bromophenol blue), and the samples were boiled for 3 min. Electrophoresis was performed as previously reported [34] using 10% polyacrylamide gels containing 0.1% sodium dodecyl sulfate. Proteins were transferred to nitrocellulose membranes, and the blots were incubated for 3 h at room temperature with primary antibodies (1:1000). The blots were then incubated with the appropriate biotinylated secondary antibodies. Reactivity was detected using the ECL (Pierce, Rockford, IL) detection system, following the manufacturer's protocol.

### Statistical analysis

Each experiment was repeated at least twice. Data are expressed as mean±standard error of the mean (SEM). Differences between means were evaluated using one-way analysis of variance (ANOVA) followed by the Bonferroni test. The accepted level of significance in all cases was p<0.001.

## Results

### The dopamine D_1_ receptor was expressed in RGC-5 cells

**Figure 1:**
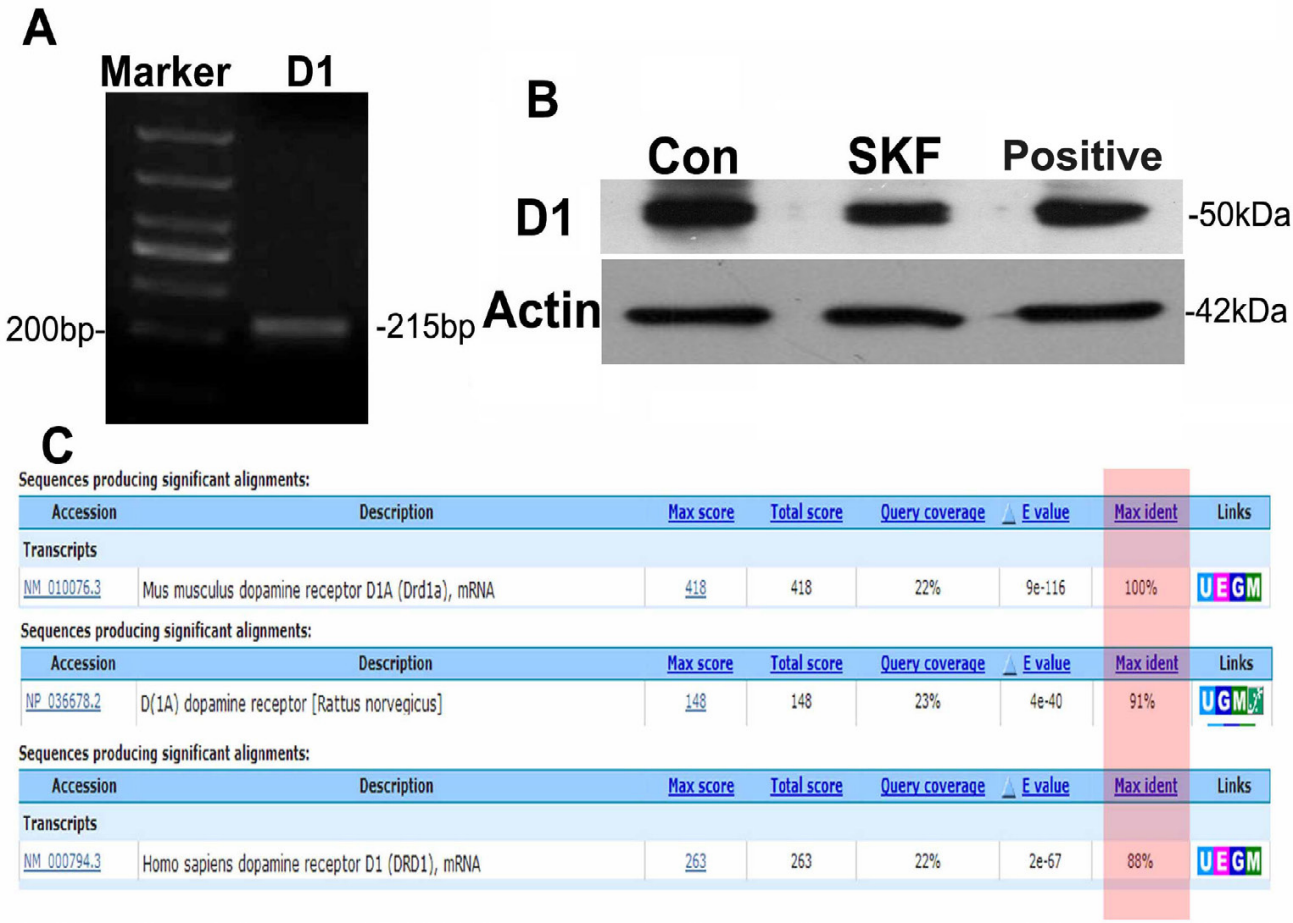
The dopamine D_1_ receptor was expressed in RGC-5 cells. **A**: Total mRNA was extracted from RGC-5 cells, and an amplicon of about 215 bp was obtained by RT–PCR using specific primers designed according to the cDNA sequence of the D_1_ receptor. **B**: D_1_ receptor protein from RGC-5 cell lysate was analyzed with western blot. Con: control, SKF: treated with 30 μM SKF83959, positive: positive control (lysate from mouse brain homogenate). A specific D_1_ receptor protein band with a molecular weight of approximately 50 kDa was detected. Treatment with SKF83959 had no significant influence on the expression of the D_1_ receptor related to the internal control, β-actin. A representative blot is shown from at least three independent experiments. **C**: Analysis and comparison of D_1_ receptor amplicons were performed using the BLAST tool (NCBI online). The maximum identification was highlighted with a pink rectangle.

Before investigating the neuroprotective action of SKF83959, the specific agonist of the D_1_ receptor, we first confirmed whether the D_1_ receptor was expressed in RGC-5 cells at the mRNA and protein levels by using RT–PCR and immunoblotting. As shown in Figure 1A, after total mRNA from RGC-5 cells was isolated and reverse transcribed into cDNA, the target sequence of about 215 bp from the D_1_ receptor cDNA was amplified with specific primers (upstream primer 5′-ATG CCA TAG AGA CTG TAA GC-3′; downstream primer 5′-GAC TAT GAC ACC GAT GTC TC-3′). The amplicon was analyzed with agarose electrophoresis and finally confirmed with DNA sequencing. Interestingly, subsequent nucleotide alignments using the BLAST server showed that the amplicon was 100% identical to the mouse D1A receptor, but only 91% and 88% homologous to rat and human D1A, respectively (Figure 1C). This indicates that RGC-5 cells were probably derived from mouse and not rat retina as previously reported [35]. Next, we further confirmed D_1_ receptor expression at the protein level with immunoblotting. As shown in Figure 1B, a specific protein band for the D_1_ receptor was detected at the appropriate molecular weight of about 50 kDa. In addition, the western blots also showed that treatment with 30 μM SKF83959 did not affect levels of the D_1_ receptor relative to the internal control, β-actin. Thus, these results suggested that the dopamine D_1_ receptor was expressed in RGC-5 cells.

### SKF83959 protected retinal ganglion cell 5 cells from H_2_O_2_-induced damage

**Figure 2:**
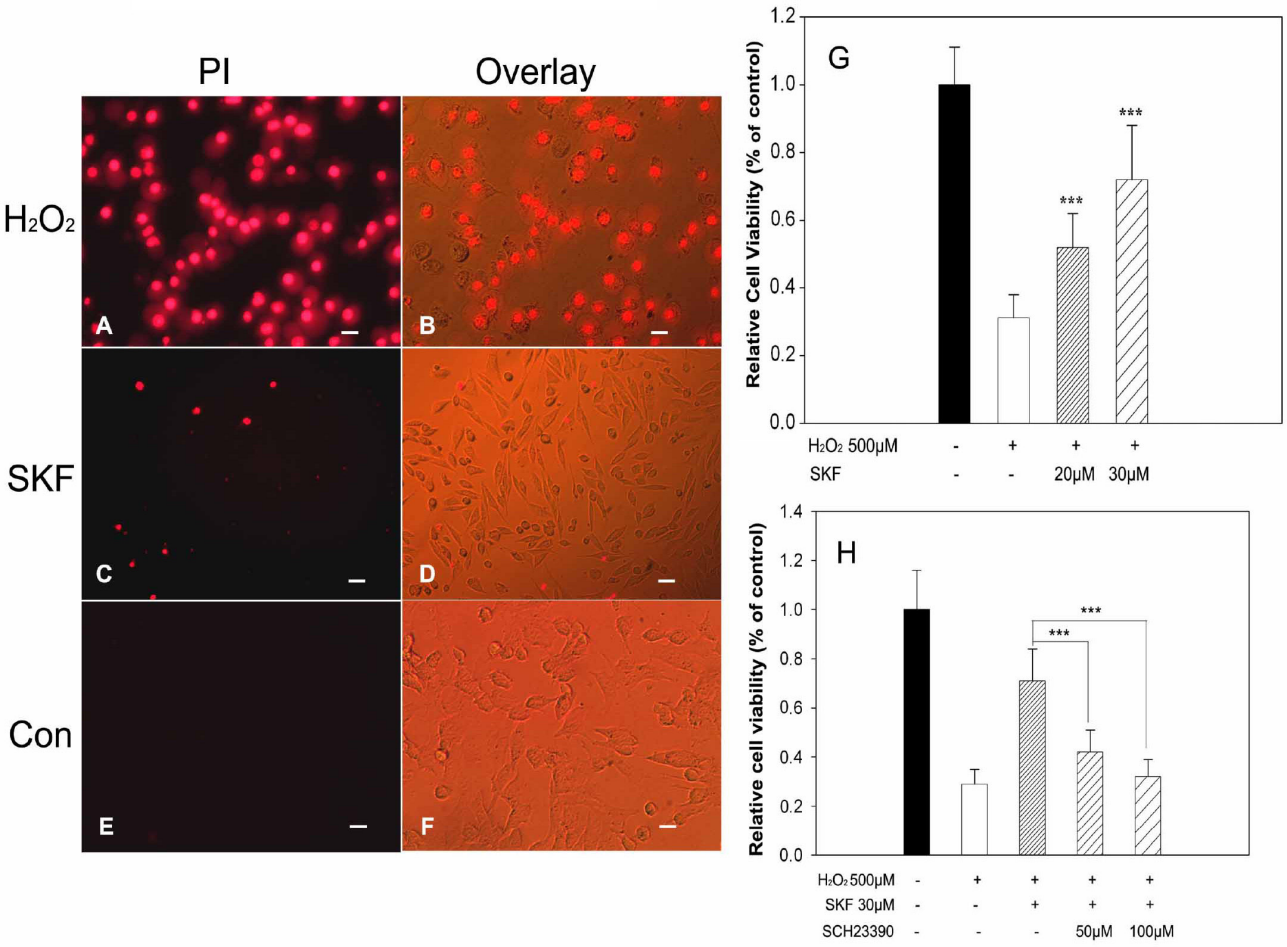
Pre-treatment with SKF-83959 attenuated H_2_O_2_-induced death in RGC-5 cells. RGC-5 cells were pre-treated with either various concentrations of SKF-83959 or vehicle for 30 min prior to and during H_2_O_2 _treatment for an additional 5 h. **A**-**F**: Cell death was detected with PI staining. The dead cells were positively tracked with PI shown in red fluorescence. Left panel: fluorescence images (PI). Right panel: merged fluorescent images with binocular convert images. **A**,** B**: RGC-5 cells were treated with 500 μM H_2_O_2_; **C**, **D**, pretreated with 30 μM SKF-83959 before 500 μM H_2_O_2_; **E**, **F**, or vehicle. Scale bar = 50 μm. **G**: Cell viability was determined via the MTT assay. Data are expressed as percentage of relative cell viability (mean±SEM, from at least three independent experiments) in relation to control treatment. ***p<0.001, compared with 500 μM H_2_O_2 _treatment. **H**: Antagonist of the D1 receptor, SCH23390, abolished the SKF-83959-induced neuroprotection. Again, viability of RGC-5 cells was determined by MTT assay. Data were obtained from at least three independent experiments and expressed as the mean±SEM. ***p<0.001, compared to the SKF-83959 treated cells.

As shown in Figure 2A–F, after treatment with 500 μM hydrogen peroxide for 5 h, massive cell loss of viability was positively detected with the PI reagent as red fluorescence observed using inverted fluorescence microscopy, whereas pretreatment with 30 μM SKF83959 30 min before 500 μM H_2_O_2_ was added effectively reduced cell death to only a few cells positively stained with PI. Consistent with the PI result, pretreatment with 20–30 μM SKF83959 significantly increased cell viability to 52.7±10.2% (20 μM) and 72.4±16.6% (30 μM) compared to 500 μM H_2_O_2_-alone treated cells (31.1±7.5%) as monitored with MTT assay (Figure 2G). Figure 2H showed that the cytoprotection of SKF83959 was remarkably blocked by the application of 50–100 μM SCH23390, a specific antagonist of the D_1_ receptor, which caused a significant reduction in cell viability back to 42.6±9.4% (50 μM) and 32.3±7.1% (100 μM). These results indicated that SKF83959 protected RGC-5 cells from oxidative stress–induced injury through a D_1_ receptor–triggered signaling pathway.

### SKF83959-induced neuroprotection involved activation of extracellular signal-regulated kinase

**Figure 3:**
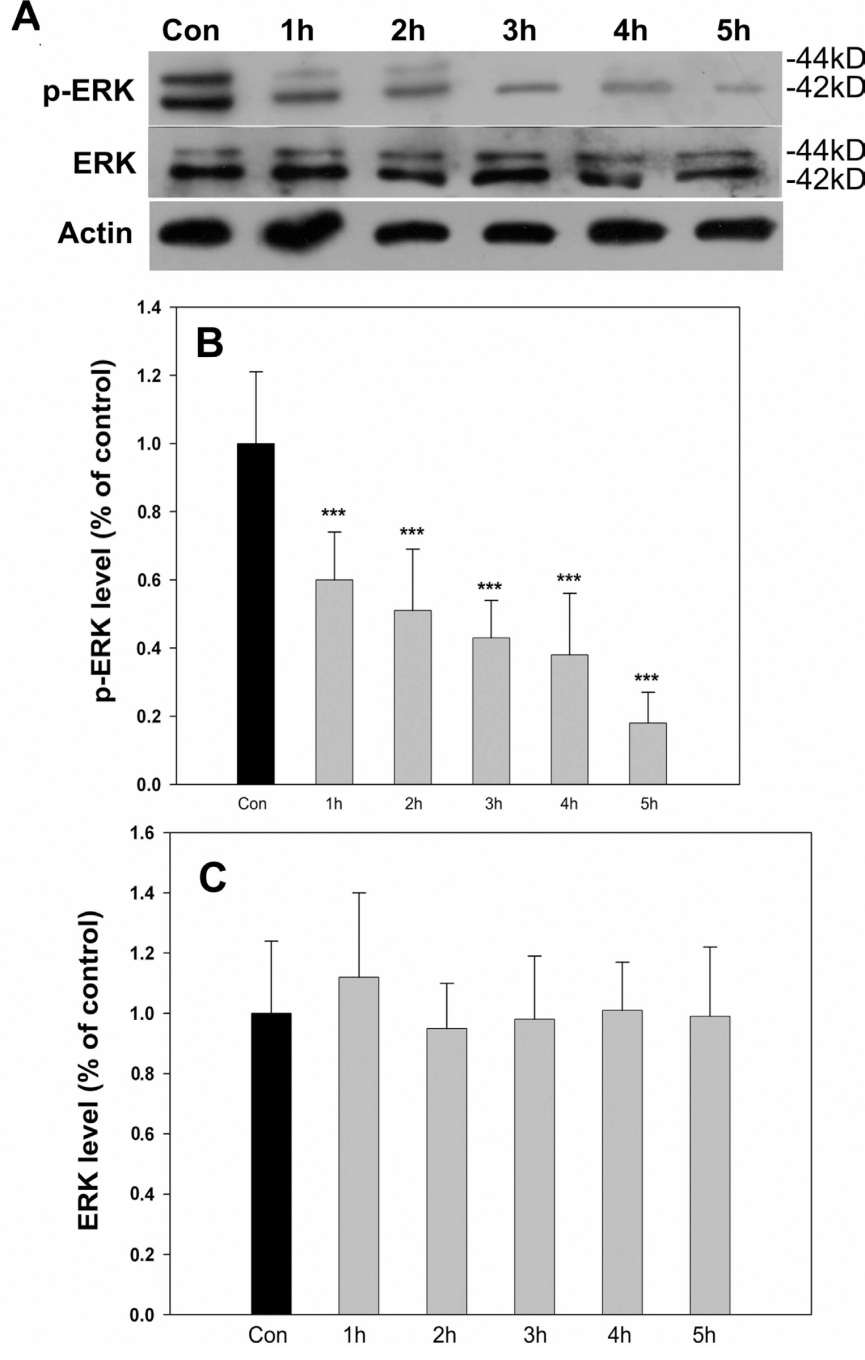
H_2_O_2_ treatment temporally decreased active levels of ERK in RGC-5 cells. RGC-5 cells were treated with various agents for designated times. Cells were then harvested and lysed for immunoblot assay **A**: 500 μM H_2_O_2_-treatment caused a gradual reduction in p-ERK levels in a time-dependent manner (1–5 h), but had little influence on total ERK compared to controls. Con: control. Each experiment was repeated at least three times, and a representative blot is shown. **B**: The reduction in p-ERK was quantitatively measured, and statistical significance was analyzed. Data were obtained from at least three independent experiments and expressed as the mean±SEM ***p<0.001, compared to controls. **C**: Total ERK levels were quantitatively measured, and statistical differences were analyzed. Data were obtained from at least three independent experiments and expressed as the mean±SEM ***p<0.001, compared to controls.

**Figure 4:**
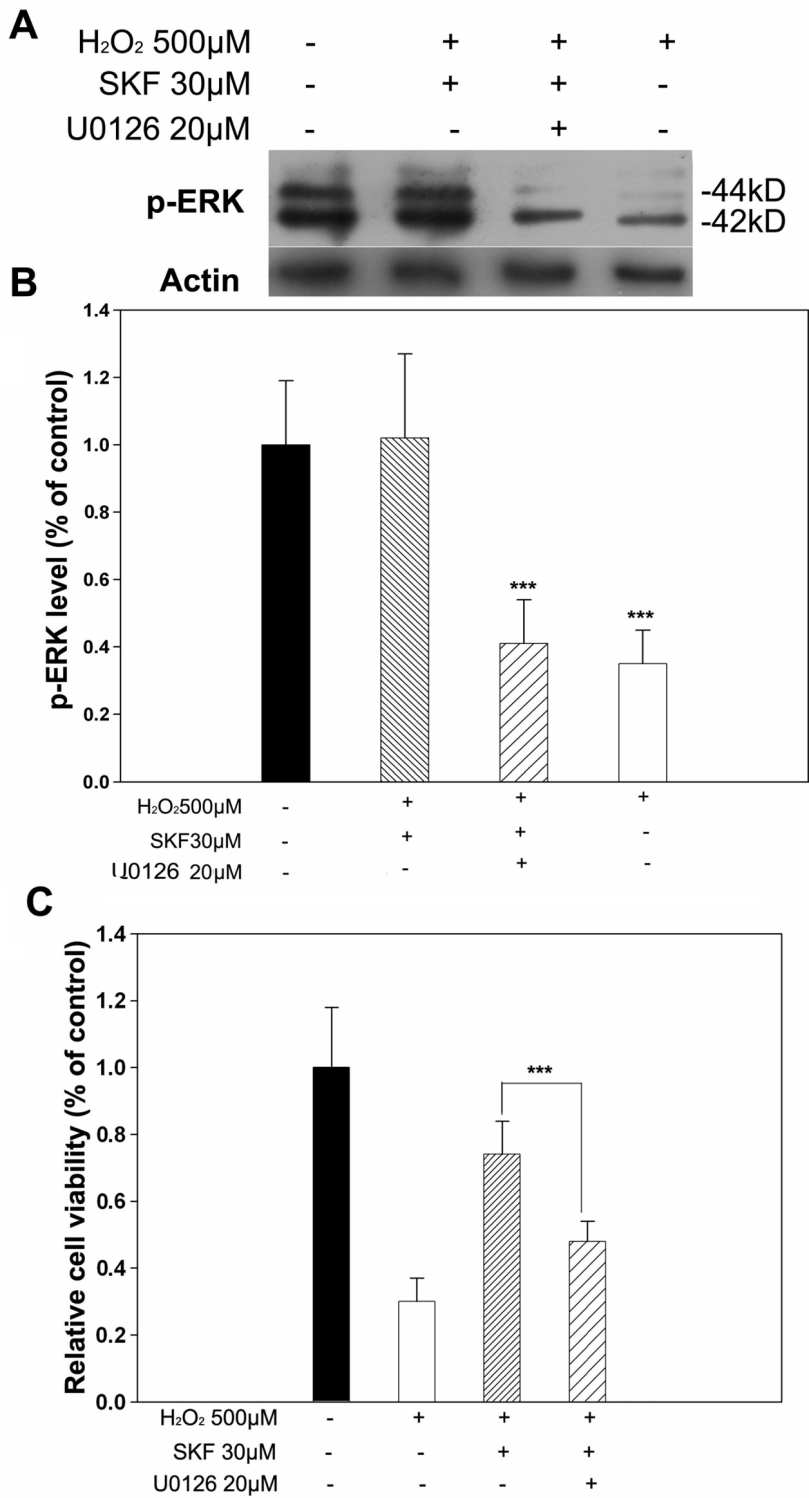
SKF83959 treatment preserved active levels of ERK in H_2_O_2_-treated RGC-5 cells. **A**: Pretreatment with 30 μM SKF83959 prevented the H_2_O_2_-induced reduction of p-ERK levels. Treatment with the specific inhibitor of MEK/ERK, 20 μM U0126, abolished the SKF83959-induced increase of p-ERK. SKF: SKF83959. Each experiment was repeated at least three times, and a representative blot is shown. **B**: The reduction of p-ERK was quantitatively measured, and statistical significance was analyzed. Data were obtained from at least three independent experiments and expressed as the mean±SEM ***p<0.001, compared to control. **C**: U0126 attenuated SKF83959-mediated neuroprotection. RGC-5 cells were pretreated with 20 μM U0126 30 min before 30 μM SKF83959 was added in the presence of 500 μM H_2_O_2_ for 5 h. Cell viability was determined with MTT assay. Data were obtained from at least three independent experiments and expressed as the mean±SEM ***p<0.001, compared to SKF83959-pretreated cells.

To demonstrate the role of ERK in SKF83959-induced neuroprotection, we first monitored any change in its active form, p-ERK, caused by H_2_O_2_ treatment. RGC-5 cells were treated with 500 μM H_2_O_2_ for various periods (1–5 h) and the active form, p-ERK, and total ERK were determined with western blot. As shown in Figure 3A, treatment with 500 μM H_2_O_2_ caused gradual reduction in p-ERK from 1 h to 5 h compared with the control cells. p-ERK consists of two protein isoforms, p-44 and p-42, detected as double bands with western blot. Hydrogen peroxide seemed to have a more dramatic effect on p-44 rather than p-42 since the protein of p-44 was undetectable by 3 h after treatment with 500 μM H_2_O_2_, whereas H_2_O_2_ treatment had little effect on total ERK. The quantitative analysis demonstrated a significant decrease in p-ERK levels from 1 h to 5 h after treatment with 500 μM H_2_O_2_ with no difference in total ERK levels (Figure 3B,C). Next, we monitored the effect of SKF83959 on p-ERK levels to see whether it regulated the activation of ERK. As shown in Figure 4A,B, treatment with 30 μM SKF-83959 30 min before hydrogen peroxide was applied led to remarkable preservation of p-ERK at relatively normal levels. However, preapplication of the specific MEK/ERK inhibitor, 20 μM U0126, attenuated the effect of SKF83959 on the p-ERK levels. To further investigate the role of ERK in SKF83959-induced neuroprotection, we monitored the action of U0126 on cell viability with MTT assay. As shown in Figure 4C, pretreatment with 30 μM SKF83959 increased cell viability by 74.7±10.2% vs. 500 μM H_2_O_2_-alone treated cells (30.2±7.5%), whereas preapplication of 20 μM U0126 significantly attenuated the protection, and cell viability was reduced to 48.2±6.3% compared to the SKF83959-pretreated cells. These results indicated that ERK was involved in the SKF83959-induced neuroprotective mechanism in H_2_O_2_-injured cells_._

### Activation of p38 MAPK was involved in neuroprotection induced by SKF83959

**Figure 5:**
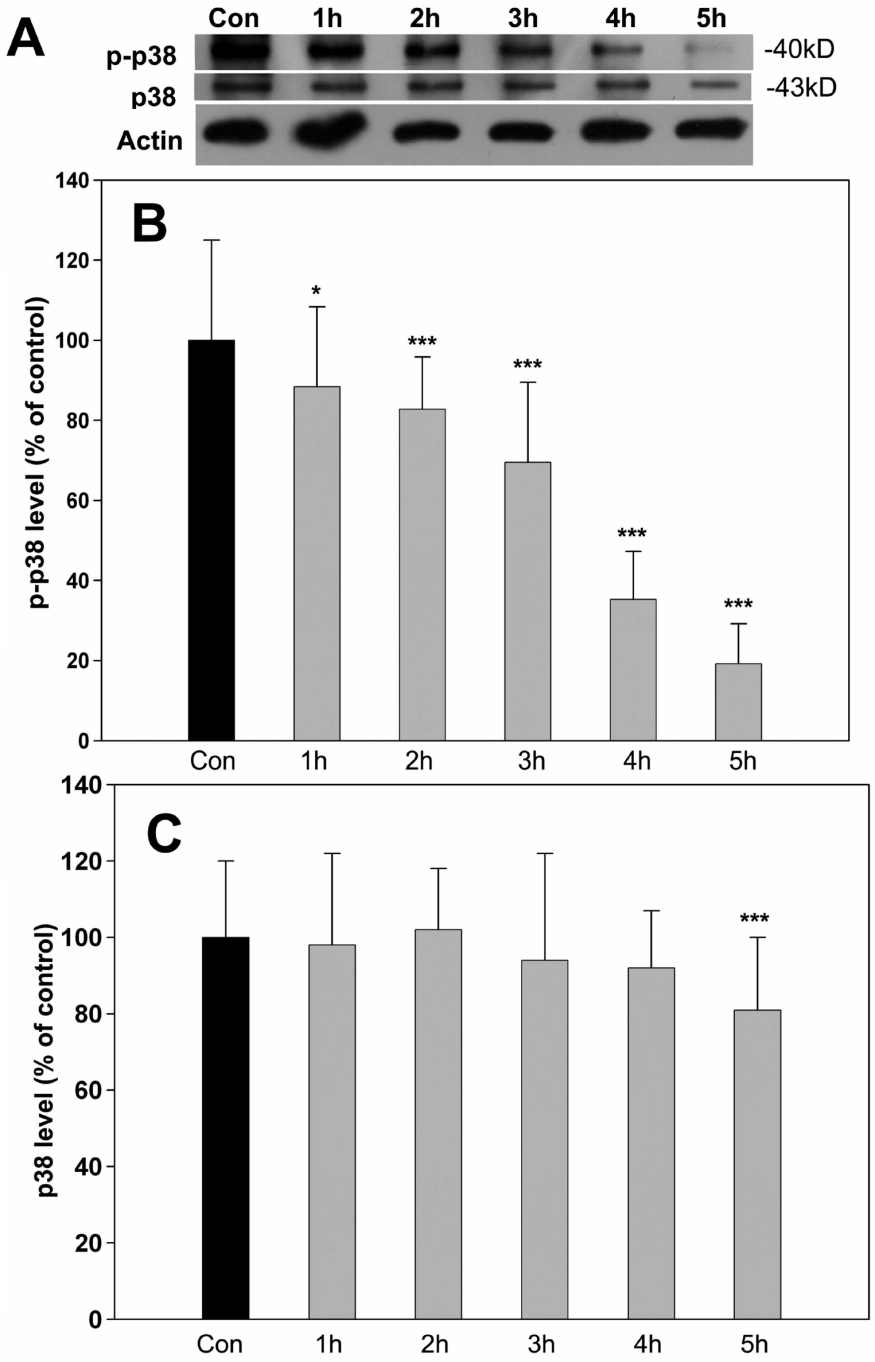
H_2_O_2_ treatment reduced levels of p-p38. RGC-5 cells were treated with 500 μM H_2_O_2_ for the designated times. Cells were then harvested and lysed for immunoblot assay. **A**: H_2_O_2_ treatment caused the gradual reduction of p-p38 in a time-dependent manner (1–5 h) but had little influence on total p38 levels compared with controls. Con: control. Each experiment was repeated at least three times, and a representative blot is shown. **B**: The reduction of p-p38 levels was quantitated and statistically analyzed. Data were obtained from at least three independent experiments and expressed as the mean±SEM ***p<0.001, compared with controls. **C**: Total p38 levels were quantitated and statistically analyzed. Data were obtained from at least three independent experiments and expressed as the mean±SEM ***p<0.001, compared with controls.

**Figure 6:**
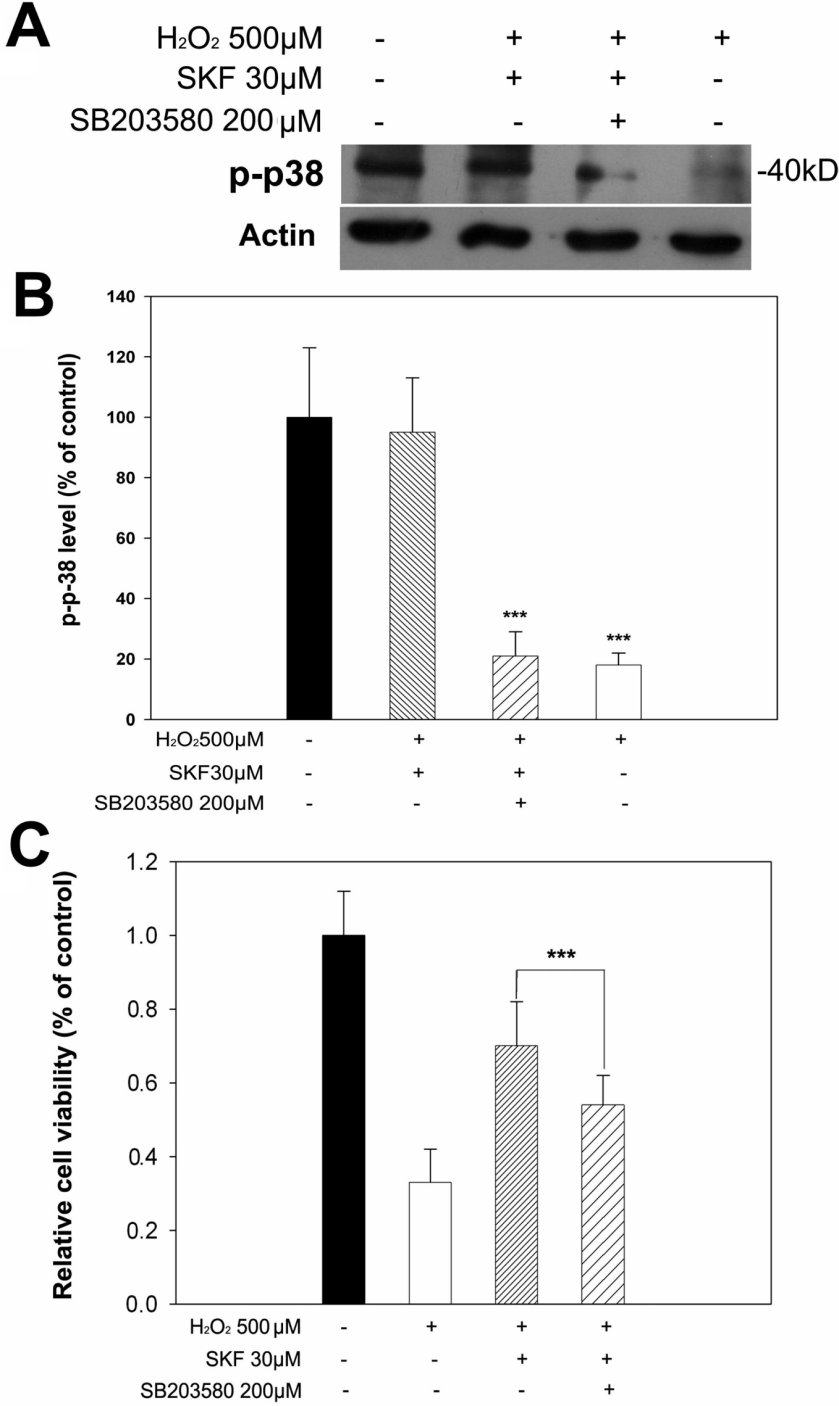
SKF83959 treatment preserved levels of active, p-p38 in H_2_O_2_-treated RGC-5 cells. RGC-5 cells were treated with various agents for the designated times. Cells were then harvested and lysed for immunoblot assay. **A**: Pretreatment with 30 μM SKF83959 prevented the H_2_O_2_-induced reduction of p-p38 levels. The specific inhibitor of p38, 200 μM SB203580, abolished the SKF83959-induced preservation of p-p38 levels. SKF: SKF83959. Each experiment was repeated at least three times, and a representative blot is shown. **B**: Levels of p-p38 were quantitated and statistically analyzed. Data were obtained from at least three independent experiments and expressed as the mean±SEM ***p<0.001, compared with controls. **C**: SB203580 attenuated SKF83959-mediated neuroprotection. RGC-5 cells were pretreated with 200 μM SB203580 30 min before 30 μM SKF83959 was added in the presence of 500 μM H_2_O_2_ for 5 h. Cell viability was determined with MTT assay. Data were obtained from at least three independent experiments and expressed as the mean±SEM ***p<0.001, compared with SKF83959-pretreated cells.

Similar to the results for active ERK levels, treatment with 500 μM H_2_O_2_ substantially decreased the levels of p-p38 in a time-dependent manner with no effect on total p38 levels as assessed with western blot (Figure 5A). Further quantitative analysis confirmed that p-p38 was significantly reduced from 1 h to 5 h compared to controls (Figure 5B), whereas total p38 levels were largely unaffected from 1 h to 4 h except a slight decrease at 5 h (Figure 5C). Importantly, pretreatment with 30 μM SKF83959 30 min before H_2_O_2_ was added largely prevented an H_2_O_2_-induced decrease in p-p38 levels (Figure 6A,B), which suggested that SKF83959 also used the p38 pathway for protection in H_2_O_2_-treated cells. Application of 200 μM SB203580, a specific inhibitor of p38, blocked the preservation effect of SKF83959 on p-p38 levels (Figure 6A,B). In addition, we assessed the role of p38 in SKF83959-induced cytoprotection with MTT assay. As shown in Figure 6C, preapplication of 200 μM SB203580 significantly blunted the SKF83959-enhanced cell viability against the H_2_O_2_ insult (from 70.7±12.8% back to 54.1±8.3%). These results indicated that activation of p38 also contributed to the neuroprotective mechanism induced by SKF83959 treatment of oxidative-stressed cells.

### c-Jun NH_2_-terminal kinase was not associated with SKF83959-induced neuroprotection

**Figure 7:**
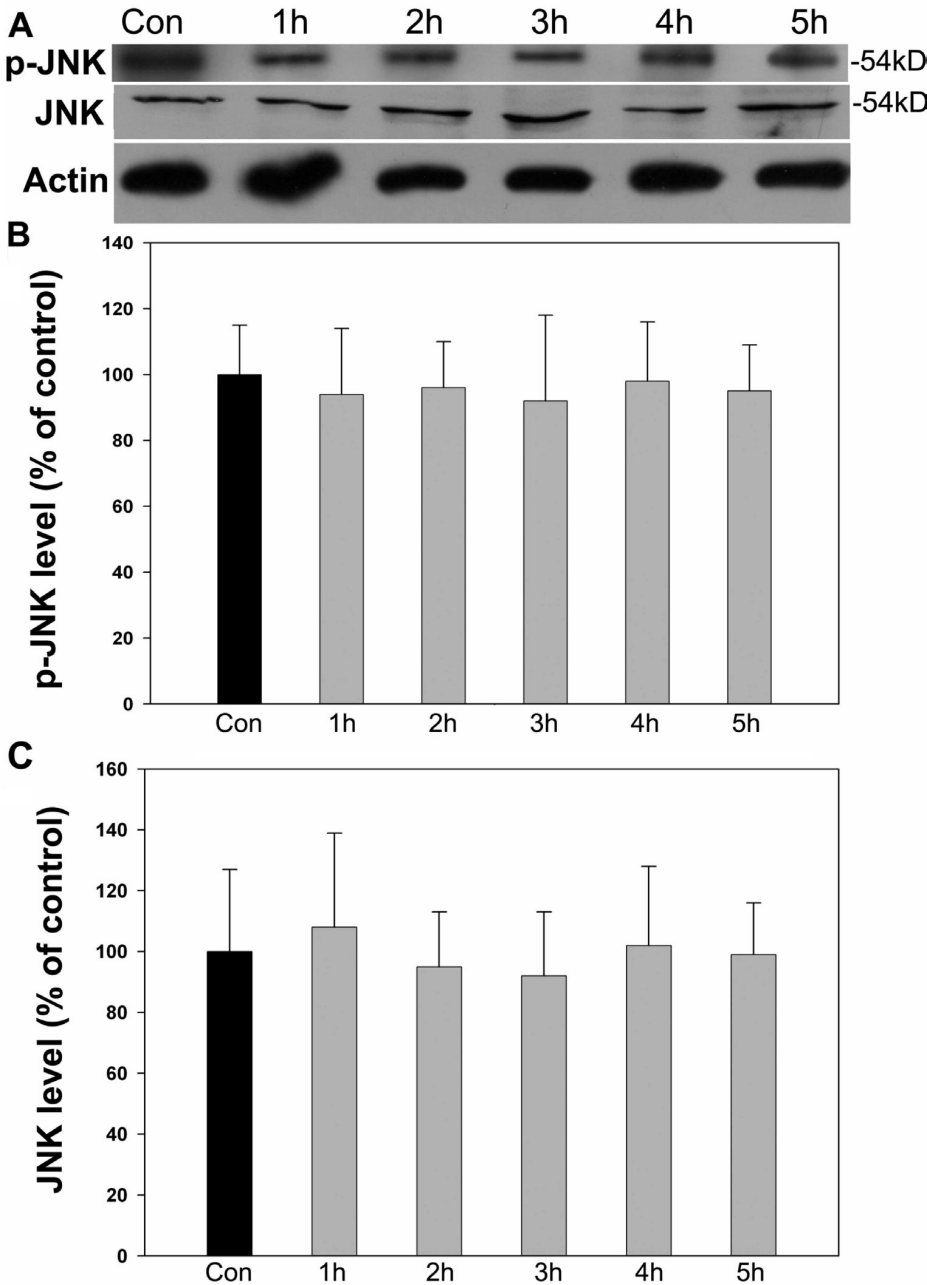
p-JNK levels were not affected by H_2_O_2_ treatment. RGC-5 cells were treated with 500 μM H_2_O_2_ for the designated times. Cells were then harvested and lysed for immunoblot assay. **A**: Treatment with 500 μM H_2_O_2_ had no significant effect on p-JNK or total JNK levels detected with western blot from 1 h to 5 h relative to the controls. Con: control. Each experiment was repeated at least three times, and a representative blot is shown. **B**: p-JNK levels were quantitated and statistically analyzed compared to controls. Data were obtained from at least three independent experiments and expressed as the mean±SEM **C**: Total JNK levels were quantitated and statistically analyzed compared to controls. Data were obtained from at least three independent experiments and expressed as the mean±SEM.

**Figure 8:**
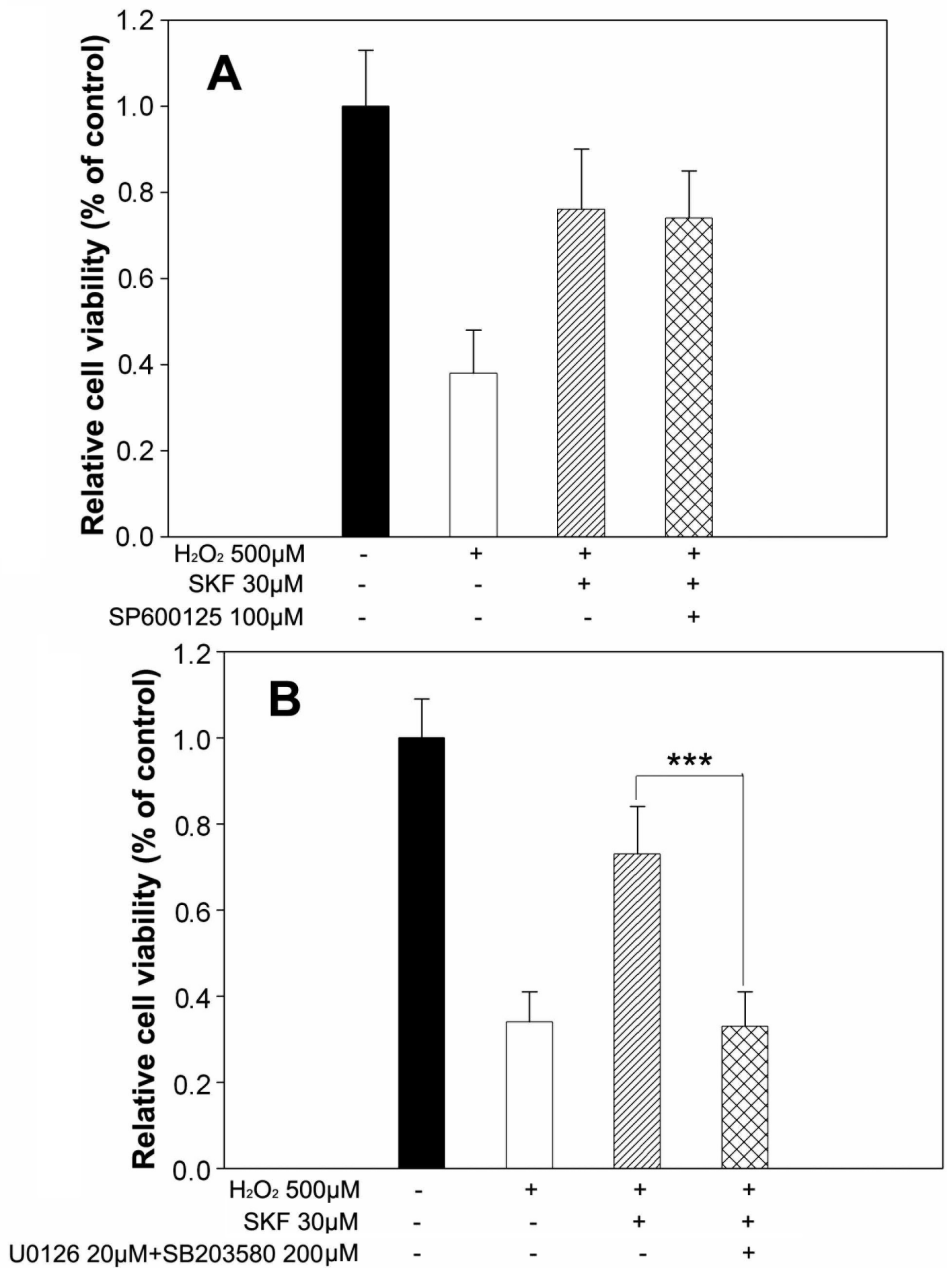
JNK activity was not associated with SKF83959-induced neuroprotection in H_2_O_2_-treated cells. **A**: The JNK inhibitor was unable to attenuate SKF83959-mediated neuroprotection. RGC-5 cells were pretreated with 100 μM SP600125 30 min before addition of 30 μM SKF83959 in the presence of 500 μM H_2_O_2_ for 5 h. Cell viability was determined with MTT assay. Data were obtained from at least three independent experiments and expressed as the mean±SEM **B**: Cotreatment with ERK and 38 inhibitors completely abolished SKF83959-mediated neuroprotection. RGC-5 cells were pretreated with 20 μM U0126 and 200 μM SB203580 30 min before 30 μM SKF83959 was added in the presence of 500 μM H_2_O_2_ for 5 h. Cell viability was determined with MTT assay. Data were obtained from at least three independent experiments and expressed as the mean±SEM ***p<0.001, compared with SKF83959-pretreated cells.

We next monitored the third member of MAPK family, JNK, at various time points after treatment with 500 μM H_2_O_2_. Different from the two other family members, applying hydrogen peroxide from 1 h to 5 h had little influence on p-JNK or total JNK levels detected with western blot (Figure 7A), and no significant changes were assessed with further statistical analysis (Figure 7B,C). Consistent with this, the specific inhibitor of JNK, 100 μM SP600125, failed to block the 30 μM SKF83959-induced cytoprotection in 500 μM H_2_O_2_-treated cells with no significant viability reduction determined with MTT assay compared with 30 μM SKF83959-treated cells (Figure 8A). However, coapplication of the ERK and p38 inhibitors remarkably reversed the cytoprotection effects of SKF83959. As shown in Figure 8B, treatment with 30 μM SKF83959 increased cell viability by 73.5±11.4% compared with the 500 μM H_2_O_2_-alone treated cells (34.2±7.6%), whereas copretreatment with 20 μM U0126 and 200 μM SB203580 caused a reduction in cell viability to 34.8±8.1%. This almost completely blocked the 30 μM SKF83959-induced cytoprotection.

## Discussion

The RGC-5 cell line has been widely used in ophthalmic research to study ocular neurodegeneration, neuroprotection, and neuroregeneration mechanisms. We appreciate that the cell line may not behave like primary RGCs in all aspects, and this is a potential limitation of our data set. However, generating primary cultures of RGCs is time-consuming and technically difficult. Therefore, for this initial study our goal was to use the RGC-5 cell line to determine the neuroprotective mechanism stimulated by SKF83959 with the expectation that the conclusions can be extrapolated to and verified in primary RGCs in a future work.

According to the first reference to RGC-5 cells previously published by Krishnamoorthy et al. [35], the RGC-5 cell line was derived from post-natal day 1 rat retinal cells by transforming with the ψ2 E1A virus and expressed thymus cell antigen 1(Thy-1), brain-3C (Brn-3C), Neuritin, the *N*-methyl *D*-aspartate (NMDA) receptor, the gamma-aminobutyric acid (GABA-B) receptor, and synaptophysin but did not express glial fibrillary acidic protein (GFAP), syntaxin 1, and 8A1, a neurofilament marker. To confirm whether RGC-5 cells expressed the D_1_ receptor, we examined the mRNA and protein levels by using RT–PCR and immunoblotting. Our results showed that a specific protein band for the D_1_ receptor was detected at the appropriate molecular weight of about 50 kDa compared with the positive control. Furthermore, we designed RT–PCR primers according to the mRNA sequence of the D_1_ receptor and obtained an amplicon with 215 bp. The sequencing results from this amplicon demonstrated that it was the targeted part of the D_1_ receptor mRNA. However, interestingly, the nucleotide alignment demonstrated that the amplicon had 100% identity with *Mus musculus* D_1_ receptor mRNA and 91% identity with* Rattus norvegicus.* The origin of the RGC-5 cell line has been somewhat controversial. Van Bergen et al. [36] recharacterized RGC-5 cells, and the study showed that the cell line was of mouse (*Mus musculus*) and not rat (*Rattus norvegicus*) origin, based on mitochondrial and nuclear DNA analyses [36]. Although not a primary focus of this study, our findings also support the notion that the RGC-5 cell line is of mouse origin. Therefore, the murine nature of the cell line should be considered in future research using RGC-5 cells to allow researchers to better select antibodies, primers, etc.

The dopamine (DA) D_1_ receptor is the most highly expressed subtype in the DA receptor family. The selective D_1_ agonist, SKF83959, belongs to the benzazepine family and possesses high affinity and selectivity. SKF83959 has a K_i_ less than 5 nM and selectivity for the D_1_ over the D_2_ receptor that is more than 3,000-fold [13]. We appreciate that the drug is not entirely specific for the D_1_ receptor but the higher reported affinity for the D_1_ receptor versus the D_2_ receptor, and the fact that the effects of the agonist were attenuated by a specific D_1_ receptor antagonist support our conclusion that the effects observed were related to the D_1_ receptor. We observed that SKF83959 treatment provided excellent neuroprotection of RGC-5 cells against oxidative stress–induced injury. Pretreatment with 30 μM SKF83959 significantly increased cell viability from 33.8% to 76.2% in hydrogen peroxide–treated cells. The cytoprotection of SKF83959 was blocked by application of 50–100 μM SCH23390, a specific antagonist of the D_1_ receptor. The retinal ganglion cells might respond to dopamine through two types of dopamine receptors. One is the D_1_ receptor based on data demonstrating detection with specific monoclonal and polyclonal antibodies. Another is a heterooligomeric D_2_–D_5_ receptor, based on calcium influx (spike firing) caused by agonists and the blockade of agonist responses by administration of antagonists. However, how retinal ganglion cells utilize D_1_ and/or D_2_–D_5_ receptors depends on various factors such as the G-proteins recruited, the duration and intensity of intracellular Ca^2+^ increases, and the subsequent signaling cascades [37].

To further investigate the molecular mechanism of SKF83959-induced neuroprotection in this study, we first assessed changes in phosphorylated, active levels in all three subfamilies of MAPK, ERK, p38, and JNK in response to H_2_O_2_-induced oxidative stress in RGC-5 cells. Our results showed that treatment with 500 μM H_2_O_2_ led to a gradual decline in p-ERK and p-p38 levels from 1 h to 5 h relative to untreated cells, but no obvious influence on p-JNK. Pretreatment with 30 μM SKF83959 resulted in a remarkable preservation of p-ERK and p-p38 levels and increased cell viability. The application of specific inhibitors of ERK and p38 significantly abrogated the cytoprotection of SKF83959 and attenuated the preservation of p-ERK and p-p38 levels. These results indicated that activation of ERK and p38 plays an important role in the SKF83959-triggered neuroprotective mechanism. ERK1/2 is phosphorylated and activated by mitogen-activated kinase/ERK kinase 1/2 [38]. This activation further leads to phosphorylation of various substrates including the 90 kDa ribosomal S6 protein kinase (Rsk), cytosolic phospholipase A2, and transcription factors such as c-Myc, NF-IL6, Tal-1, Ets-2, and Elk [39]. The upregulation of gene transcription results in increased expression of antiapoptotic Bcl-2 family members and inhibitor of apoptosis proteins (IAPs) [40]. For p38, it is generally phosphorylated and activated by MEK3/6 in response to variety environmental stresses and inflammatory cytokines. Antiapoptotic roles of p38 have been described in endothelial cells exposed to anoxia-reoxygenation [41], differentiating neurons [42], and activated macrophages [43]. The role of p38 in preventing apoptosis is similar to that described for ERK involving regulation of protective transcription factor activity [44]. In our study, another subfamily member of MAPK, JNK, appeared to play a less important role in the molecular mechanism of oxidative stress–induced death and SKF83959-caused cytoprotection in RGC-5 cells since no significant change in active p-JNK levels was found, and its specific inhibitor failed to block SKF-induced neuroprotection. The cells appeared to have basally high levels of phosphorylated, active forms of the kinases in control conditions. One explanation for this phenomenon is that basal activity of the enzymes might be particularly high due to trophic or mitogenic stimuli during the culture conditions.

To our knowledge, this study is the first demonstration that the dopamine D_1_ receptor is expressed in RGC-5 cells, and its amplicon from mRNA suggests RGC-5 cells are from mouse (*Mus musculus*) and not rat (*Rattus norvegicus*) origin, which is consistent with a previous study [36]. The agonist of the D_1_ receptor, SKF-83959, effectively rescued RGC-5 cells against hydrogen peroxide–induced injury, and ERK and p38 play important roles in the molecular mechanism of neuroprotection. Our results may enhance the current understanding of the molecular mechanisms of retinal ganglion cell death in diseases associated with oxidative stress and provide a basis for future studies to develop neuroprotective drugs with the dopamine D_1_ receptor as a new therapeutic target.
